# Asthma therapy concepts through the ages 

**DOI:** 10.5414/ALX02445E

**Published:** 2024-01-12

**Authors:** Marek Lommatzsch

**Affiliations:** Department of Pneumology, Rostock University Medical Centre, University of Rostock, Rostock, Germany

**Keywords:** asthma, symptom prevention, DMAADs, remission

## Abstract

The development and approval of DMAADs (“disease-modifying anti-asthmatic drugs”), in particular inhaled steroids (alone or in combination with long-acting bronchodilators), biologics and modern allergen immunotherapies, has fundamentally changed the asthma therapy concept from symptom control to symptom prevention. This concept is linked to the new asthma treatment goal of asthma remission: long-term absence of symptoms (good asthma control), absence of exacerbations, and stable lung function, without the use of systemic steroids for asthma therapy. Three types of asthma remission are distinguished: spontaneous remission (e.g., childhood asthma), remission “off treatment” (e.g., after successful allergen immunotherapy), and remission “on treatment” (e.g., during inhaled therapy or biologic therapy). A treat-to-target approach is used, as in rheumatoid arthritis or chronic inflammatory bowel disease: The goal is to achieve asthma remission, through individually tailored treatment with highly effective drugs with minimal side effects. However, this requires precise phenotyping of the patient, including detailed history taking, pulmonary function diagnostics, allergological diagnostics, and measurement of type 2 biomarkers.

## History of asthma therapy: symptom control as the goal 

Until well into the 20^th^ century, the aim of asthma therapy was the acute treatment of airway obstructions: Doctors waited for symptoms to appear, reacted to these symptoms, and were happy if they could control them to some extent. Until the middle of the 20^th^ century, this was done with sympathomimetics (e.g., ephedrine preparations or inhaled adrenaline), anticholinergics (inhaled scopolamine, with so-called “asthma cigarettes”), or methylxanthines (oral caffeine or theophylline) [1]. Although systemic glucocorticoids (oral, intravenous, or intramuscular) and short-acting beta-agonists (SABA) (initially isoproterenol and orciprenaline, later more beta-2-receptor-selective drugs, such as salbutamol or fenoterol) available since the 1950s and 1960s were a significant pharmacological advance, these drugs were still primarily used to treat acute asthma symptoms. These drugs were neither intended nor expected to prevent symptoms. In addition, both systemic glucocorticoids and SABA monotherapies had severe side effects and were associated with increased mortality [2]. Cromones (first available in 1967) had few side effects, but also few anti-asthmatic effects. Thus, until well into the 1970s, long-term asthma therapy consisted of medications which were used to constantly treat acute symptoms (“fire extinguishers”) and which had either considerable side effects (e.g., prednisolone) or little effect (e.g., cromones) [2]. The “advantage” from the point of view of general asthma care was the uniform and clear treatment regimen (SABA, prednisolone, and theophylline): the patients did not have to be phenotyped, there were “standard fire extinguishers” that applied to all patients. This lack of need for more detailed patient characterization and the simple portfolio of standard medications were the secret of the very successful and sustainable implementation of these treatment recommendations in asthma care. The anchoring in the collective memory of physicians was so strong and lasting that it still resonates in non-specialized care (some colleagues still live the concept of symptom control, as the acute medications “help well”). At the same time, the deep-seated fear of cortisone, which can torpedo modern therapy with low-dose inhaled corticosteroids (ICS) (typical anxious question from patients: “Is there cortisone in the spray?”), remains in the collective lay memory as an echo of deleterious systemic steroid therapies. 

## Changes in asthma therapy: symptom prevention as a goal 

The asthma treatment concept has changed fundamentally over the last 50 years. The new treatment concept is based on the principle of symptom prevention through targeted therapy with modern anti-inflammatory drugs [2, 3]. This paradigm shift was made possible in particular by the availability of modern ICS (either as low-dose monotherapy or in combination with long-acting beta-agonists (LABA), and/or long-acting muscarinic antagonists (LAMA)), and the availability of biologics and modern allergen immunotherapies (AIT). These drugs (analogous to disease-modifying antirheumatic drugs (DMARDs) in rheumatology) are summarized under the overarching term disease-modifying anti-asthmatic drugs (DMAADs) [2]. DMAADs are not only highly effective, but also have few side effects. The concept of *preventing symptoms *(e.g., through the early use of ICS in mild forms of asthma: either as ICS/formoterol as-needed therapy or as low-dose ICS maintenance therapy) is therefore closely linked to the concept of *preventing side effects *(e.g., by prioritizing biologics over systemic steroids in severe asthma). Instead of side effects (“collateral damage”), DMAAD therapy can even lead to an improvement in typical asthma comorbidities (“collateral efficacy”): e.g., biologics used to treat severe asthma can also have beneficial effects on chronic sinusitis with nasal polyps, urticaria, atopic dermatitis, or vasculitis [4]. It is important to note that the concept of symptom prevention does not only refer to long-term drug therapy. On the one hand, allergen immunotherapy as an important DMAAD is a temporary treatment (typically over 3 years) [5, 6], on the other hand, on-demand therapy in asthma is also changing: away from pure on-demand bronchodilation (reliever) towards on-demand therapy with an anti-inflammatory component (anti-inflammatory reliever (AIR)) [7]. In fact, increasing evidence indicates that even in mild forms of asthma, on-demand therapy with a fixed combination of ICS and a fast-acting beta-agonist (FABA) (such as formoterol or salbutamol: ICS/FABA on-demand therapy) is superior to on-demand therapy with a SABA alone (such as salbutamol) [8]. Both the international guidelines of GINA [9] and the ERS [8] as well as the new German asthma guideline for respiratory specialists [10] therefore unanimously recommend that ICS/FABA on-demand therapy is preferable to SABA-only therapy in patients with mild forms of asthma. The general concept of treatment recommendations is therefore a phenotype-specific, anti-inflammatory DMAAD therapy, moving towards clever “fire prevention” and away from constant “fire extinguishing”. 

## Asthma remission as a new treatment goal 

**Figure 1.**
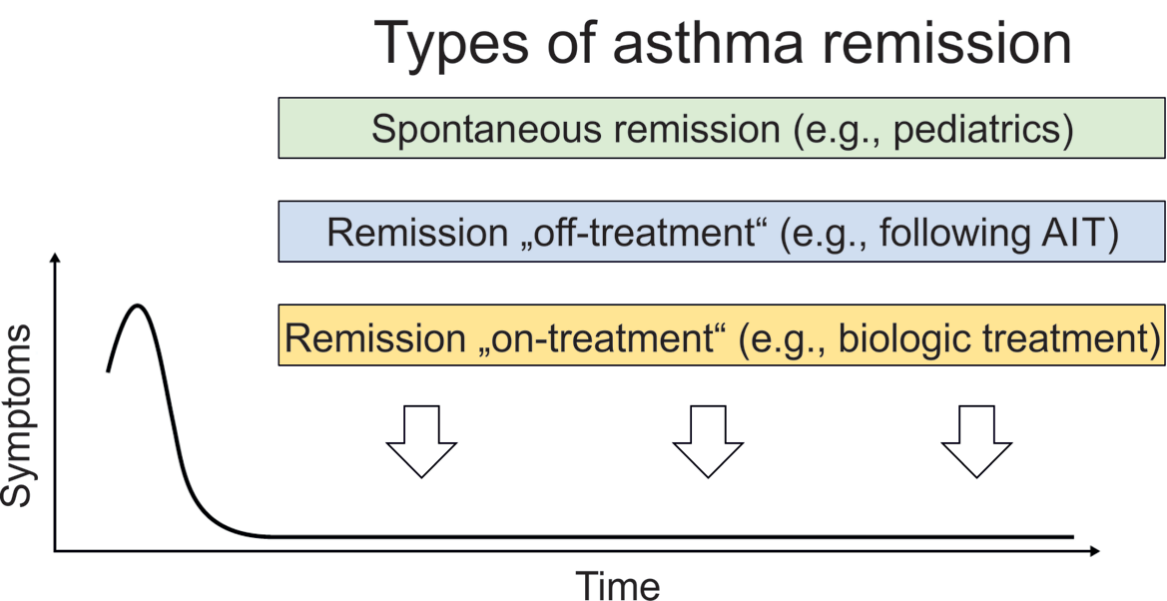
Types of asthma remission.

The treatment goal of remission, in conjunction with the treat-to-target concept (target = remission) has long been established in other chronic inflammatory diseases such as rheumatoid arthritis [11, 12], polymyalgia rheumatica [13], systemic lupus erythematosus [14], chronic inflammatory bowel diseases [15], and ANCA-associated vasculitis (such as eosinophilic granulomatosis with polyangiitis (EGPA), which is closely associated with asthma) [16]. These remission concepts are based on the assumption that remission can also occur during anti-inflammatory therapy (remission on treatment). Previously, the concept of asthma remission was limited to spontaneous remission (especially in pediatrics) or remission after treatment (especially in allergology, after allergen immunotherapy) (Figure 1). The concept of asthma remission on treatment was previously rejected by many respiratory physicians, as it was not seen as a form of disease modification (in contrast to all other specialties of internal medicine) and, moreover, it could not be imagined that patients with certain forms of asthma (especially patients with severe asthma) could ever achieve this goal [17]. However, with the introduction of highly effective biologics for the treatment of severe asthma and the increasing number of permanently symptom-free patients on this therapy [18, 19, 20, 21], these views have changed. Definitions of asthma remission independent from the treatment status have been proposed and linked to a long-term vision (criteria must be met for at least 12 months) [2, 22, 23, 24]. Three elements are included in all definitions: 1. no occurrence of asthma exacerbations, 2. no need for systemic steroids for asthma therapy, and 3. minimal or absent asthma symptoms. In addition, most definitions require long-term stable lung function as the 4^th^ remission criterion [2, 26]. 

## The new specialist asthma guideline 2023 as a pioneer 

**Figure 2.**
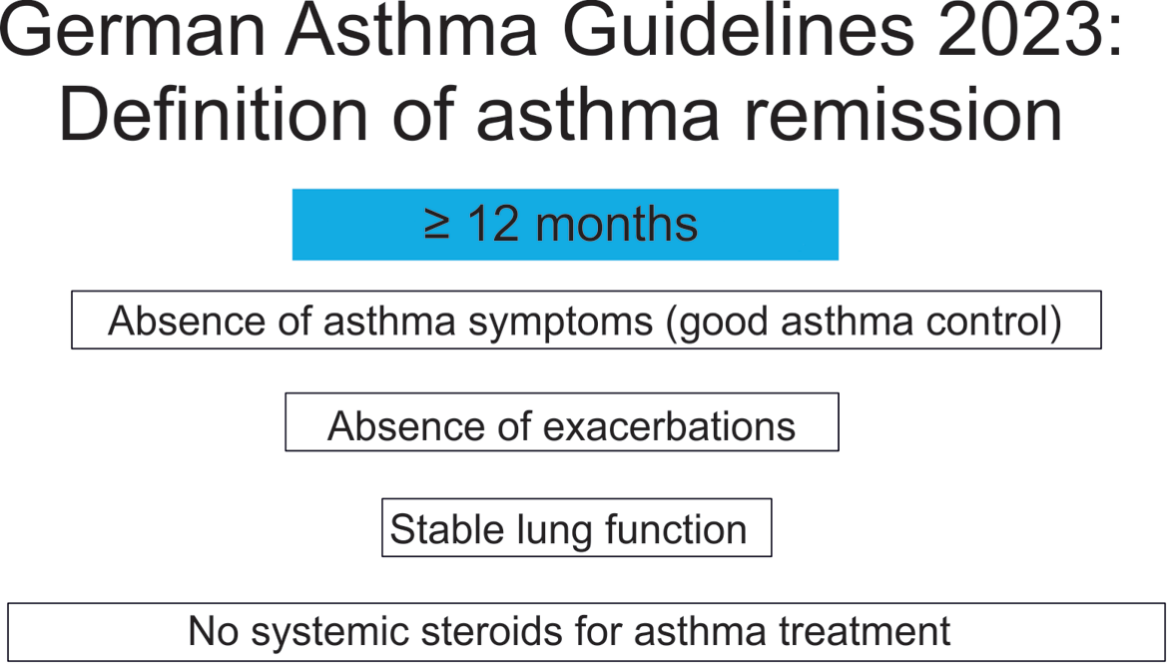
Definition of asthma remission according to the 2023 guideline [10].

The new German asthma guideline for respiratory specialists 2023 [10] was the first guideline worldwide to announce clinical asthma remission as an asthma treatment goal and proposed 4 criteria (Figure 2). In the meantime, the Spanish, Japanese, and Italian asthma guidelines have followed this example, with slight nuances in the definition criteria [26]. For example, the German and Spanish guidelines refer to the “absence of asthma symptoms”, while the Japanese (Asthma Control Test (ACT): at least 23 points) and Italian guidelines (ACT: at least 20 points) use specific ACT thresholds. This shows that there is still no international consensus on the exact clinical remission criteria [26]. It is also currently being discussed whether the term biological (immunological) remission (clinical remission plus absence of signs of inflammation) should be introduced in addition to clinical remission [26]. 

## The future of asthma therapy 

Step-up escalation schemes of asthma therapy will become less important, as the DMAADs will no longer be built on each other hierarchically according to a fixed canon, but will be selected on a phenotype-specific basis. There will be a non-hierarchic landscape of treatment options in which DMAADs are individually combined with each other and used variably over time. The asthma therapy of the future will consist of two phases – an initial phase of remission induction (higher doses, typically a combination of several DMAADs) and a second phase of remission maintenance (as few DMAADs as possible, in the lowest possible dose) [3]. The long-term goal is to achieve remission maintenance with as few side effects as possible. It is clear that the precise phenotyping of patients with asthma (from a detailed medical history to the measurement of biomarkers [25]) is an essential prerequisite for inducing and maintaining remission. Finally, the detection and treatment of typical comorbidities is also of fundamental importance [25]. 

## Funding 

None. 

## Conflict of interest 

M. Lommatzsch has received research support from DFG, GSK, Astra Zeneca as well as honoraria for lectures and consultancy work from ALK, Allergopharma, Astra Zeneca, Bencard, Berlin-Chemie, Boehringer-Ingelheim, Bosch, Chiesi, Circassia, GSK, HAL Allergy, Janssen-Cilag, Leti, MSD, Mundipharma, Novartis, Nycomed/Takeda, Sanofi, Stallergenes, TEVA, and UCB.
